# Editorial: Physical exercise and metabolic health in children and adolescents

**DOI:** 10.3389/fmed.2025.1558522

**Published:** 2025-01-28

**Authors:** Stevo Popovic, Gianpaolo De Filippo, Noelia Gonzalez-Galvez

**Affiliations:** ^1^Faculty for Sport and Physical Education, University of Montenegro, Niksic, Montenegro; ^2^Balkan Institute of Science and Innovation, University of Montenegro, Podgorica, Montenegro; ^3^K-CLUB, Korea University, Seoul, Republic of Korea; ^4^Assistance Publique-Hôpitaux de Paris, Hôpital Robert-Debré, Service d'Endocrinologie et Diabétologie, Paris, France; ^5^Groupe de Recherche en Médecine et Santé des Adolescents (GRMSA), Paris, France; ^6^Facultad de Deporte, UCAM Universidad Católica de Murcia, Murcia, Spain

**Keywords:** physical activity, metabolic health, childhood obesity, exercise interventions, children and adolescents

## Introduction

The alarming rise in childhood obesity and related metabolic disorders represents a pressing global health challenge, with over 340 million children and adolescents aged 5–19 categorized as overweight or obese, according to the World Health Organization (1). This epidemic underscores the critical need for evidence-based strategies to promote metabolic health through physical activity. Regular engagement in structured exercise interventions, habitual physical activity, and innovative approaches has improved youth insulin sensitivity, lipid profiles, and overall metabolic function (2, 3). By examining diverse perspectives and methodologies, this Research Topic provides actionable insights into developing effective interventions and policies to combat this pervasive issue.

## Contributions to the field

Nine compelling articles addresses the multifaceted relationship between physical activity and metabolic health, exploring various aspects of exercise science, from metabolic biomarkers to psychosocial outcomes. Together, they provide a comprehensive overview of current research and practical applications in the field through four thematic areas: (1) Exercise and metabolic biomarkers, (2) Exercise interventions and obesity management, (3) Psychosocial and cognitive benefits of physical activity, and (4) Policy recommendations for physical activity of the youngsters.

## Exercise and metabolic biomarkers

In this Research Topic, Vasileva et al. highlight the effects of neuromuscular training on metabolic biomarkers (e.g., salivary high molecular weight adiponectin) and physical fitness parameters, linking exercise interventions to measurable physiological outcomes. These findings align with broader evidence that exercise modulates key metabolic pathways, reducing the risk of type 2 diabetes and cardiovascular diseases (4, 5).

## Exercise interventions and obesity management

Several articles address the critical challenge of childhood obesity and explore strategies for its management. Aniśko et al. emphasized body mass composition as a key predictor of overweight and obesity, aligning with broader efforts to prevent and manage obesity through targeted interventions and regular monitoring. Their study revealed significant differences in body composition between ballet school students and their peers in traditional schools, suggesting that specialized physical training, such as ballet, can notably influence these metrics in children and adolescents. Similarly, Deng and Wang demonstrated that high-intensity interval training (HIIT) is an effective intervention for improving cardiorespiratory fitness in children and adolescents with overweight or obesity. Morgado et al. further highlighted the benefits of combining exercise with nutrition education to enhance children's health and wellbeing. Additionally, Ju et al. explored virtual reality-based exergaming as a structured intervention to improve cardiopulmonary fitness in children, aligning with targeted exercise strategies for managing obesity. Together, these studies underscore the importance of multi-component interventions in effectively addressing obesity in youth populations.

## Psychosocial and cognitive benefits of physical activity

Beyond physical health, exercise has equally significant psychosocial and cognitive benefits. Zhao et al. systematically reviewed the health impacts, including psychosocial and cognitive outcomes, of adhering to movement guidelines encompassing physical activity, sedentary behavior, and sleep. Additionally, Joung et al. examined how perceived enjoyment and exercise commitment influence behavioral intentions, emphasizing the psychological and social dimensions of physical activity participation among adolescents. These findings highlight the holistic advantages of exercise, extending its impact on mental wellbeing and cognitive development.

## Policy recommendations for physical activity of youngsters

The final section of this Research Topic emphasizes the necessity of systemic approaches to encourage physical activity among children and adolescents. Ruan and Tang provide longitudinal data and insights into changes in adolescent physical fitness, which can inform policies to improve youth activity levels and health outcomes. At the same time, Liao et al. offer insights that could inform guidelines promoting outdoor activities to enhance children's vision health. These findings are vital for shaping public health policies integrating physical activity into daily routines and institutional frameworks.

## Conclusion

The nine articles on this Research Topic collectively reinforce physical activity's essential role in enhancing children's and adolescents' metabolic and psychosocial health. Future research should explore the long-term impacts of different exercise types and intensities and the integration of new technologies to promote activity. On the other hand, policymakers must prioritize equitable access to safe environments for physical activity, incorporating these into educational and community frameworks. Such actions will foster healthier generations and mitigate the burden of metabolic disorders worldwide.
